# Structural Integrity Enhancement and Sustainable Machining Process Optimization for Anti-Lock Braking System Hydraulic Valve Blocks

**DOI:** 10.3390/ma18235287

**Published:** 2025-11-24

**Authors:** Alexandru-Nicolae Rusu, Dorin-Ion Dumitrascu, Adela-Eliza Dumitrascu

**Affiliations:** 1 Department of Manufacturing Engineering, Transilvania University of Brasov, 5 Mihai Viteazul, 500036 Brasov, Romania; alexandru.rusu@unitbv.ro (A.-N.R.); dumitrascu_a@unitbv.ro (A.-E.D.); 2 Department of Automotive and Transport Engineering, Transilvania University of Brasov, 1 Politehnicii, 500036 Brasov, Romania

**Keywords:** automotive safety systems, computational fluid dynamics, structural analysis, finite element analysis, topological optimization workflows, process performance

## Abstract

This paper presents an in-depth study on the structural integrity enhancement and machining process optimization of Anti-lock Braking System (ABS) hydraulic valve blocks, focusing on the transition from the MK60 to the MK100 design. The research combines finite element analysis (FEA), topology optimization, fixture redesign, and coolant technology improvements to achieve significant performance, productivity, and sustainability gains. The MK100 exhibits a mass reduction of 31.6%, an increase in tensile strength by 29.2%, and a fatigue life extension of 35% compared to the MK60. Pressure losses have been reduced by 38.8%, improving braking system responsiveness. On the manufacturing side, fixture redesign increased production capacity from 240 to 480 parts per shift while reducing cycle time from 16 min to 8 min per lot. The transition from a semi-synthetic emulsion coolant (AquaCut EM-X45) to a bio-based oil (BioLube AL-2200) extended coolant replacement intervals from six months to two years, reduced tooling costs, and increased tool life by 25%. These findings demonstrate the feasibility of integrating computational design methods with advanced machining strategies to achieve measurable mechanical and economic benefits in the automotive industry.

## 1. Introduction

The continuous advancement of automotive safety systems has significantly transformed the field of vehicle dynamics and braking technology. Anti-lock Braking Systems (ABSs) represent one of the most critical safety features, ensuring optimal tire–road friction and vehicle stability during braking events [1]. Over the last decades, several generations of ABS units have evolved, from early electromechanical modulators to highly integrated mechatronic control units with adaptive modulation capabilities [2]. Recent studies have emphasized the importance of optimizing hydraulic valve blocks, which remain the mechanical core of ABS units, in order to improve performance, weight efficiency, and manufacturing sustainability [3,4,5].

Earlier research focused on pneumatic and hydraulic control dynamics demonstrated that the accuracy of valve actuation and hydraulic response time significantly influences braking distance and stability control [1,6]. Computational models of electro-hydraulic valves have shown that internal flow distribution and pressure modulation stability are highly sensitive to the geometric configuration of the manifold and to manufacturing tolerances [7,8]. Consequently, valve block optimization has become an important topic in modern mechanical design, integrating computational fluid dynamics (CFD), finite element analysis (FEA), and topological optimization workflows to balance performance with manufacturability [2,9,10]. Additionally, several studies show that failures of flexible brake hoses caused by aging, thermal cycling, or internal layer delamination can also contribute to hydraulic brake system malfunctions, highlighting the importance of a holistic understanding of all components involved in pressure modulation.

The structural behavior of aluminum valve blocks under high cyclic loading conditions has also been extensively studied. According to Shao et al. [11], the adoption of aluminum–lithium (Al–Li) alloys can provide superior specific strength and fatigue resistance compared to conventional 6000-series alloys. Their precipitation-hardened microstructure allows for thinner wall sections and improved durability, which are key requirements for compact ABS housings subjected to hydraulic pulsations. This material innovation aligns with the broader objective of vehicle mass reduction without compromising safety [12].

However, the optimization of ABS components extends beyond material substitution. Manufacturing and process-related parameters significantly affect overall system performance and lifecycle costs. Traditional machining of ABS blocks, typically performed on 4-axis CNC centers, involves complex clamping systems and long machining cycles. Cooling and lubrication strategies, based on semi-synthetic emulsions, introduce limitations in tool life and environmental impact [13,14]. The introduction of bio-based lubricants and high-efficiency milling (HEM) toolpaths, as recommended in sustainable machining frameworks [15,16], can improve productivity while reducing chemical waste and maintenance requirements.

From an industrial and environmental perspective, the integration of Industry 4.0 principles–such as digital twin modeling, sensor-based process monitoring, and life-cycle analysis (LCA)—enables advanced optimization of both design and production processes [17,18]. Research on sustainable machining fluids [13,15] and energy-efficient manufacturing methods [19,20] indicates that mechanical performance, cost efficiency, and ecological impact can be optimized simultaneously through multi-objective design methodologies.

This research builds upon those foundations by redesigning and optimizing the ABS hydraulic valve block from the legacy MK60 generation to a new MK100 configuration.

The main goals are to:

- Reduce mass and hydraulic pressure loss;
- Increase mechanical stiffness and fatigue life;
- Improve production efficiency and coolant sustainability;
- Maintain full mechanical compatibility with existing ABSs.

The design follows a multi-physics methodology combining FEA, CFD, and topological optimization, validated experimentally through prototype testing and machining process analysis. By introducing new materials, optimized fixture systems, and bio-based cooling technology, this study demonstrates a pathway toward high-performance, sustainable hydraulic component design suitable for industrial implementation [21,22].

**Figure 1:**
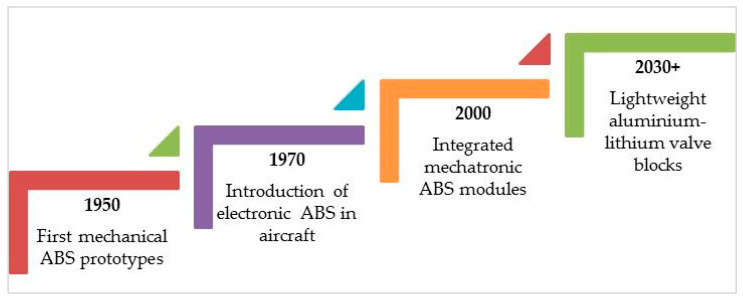
Evolution timeline of ABS technology from early mechanical systems to predictive, self-healing designs.

As shown in Figure 1, the development of anti-lock braking systems (ABSs) has evolved from early mechanical designs to intelligent, self-healing structures.

## 2. Materials and Methods

This study establishes an end-to-end design and validation workflow to retrofit legacy brake systems with a new aluminum-alloy ABS valve block (MK100) derived from an archived MK60 hydraulic schema and to demonstrate quantifiable gains in mass, mechanical resistance, fatigue life, hydraulic loss, and unit production cost. The workflow integrates reverse engineering, multi-physics modeling, topology optimization under manufacturability constraints, fixture and process planning redesign for high-throughput machining, coolant strategy transition to bio-based lubrication, and laboratory validation, while maintaining compatibility with existing vehicle hydraulic architectures and control logic. The methodology was conceived to be repeatable, auditable, and transferable to other manifold-type hydraulic actuators and was defined a priori before any design decisions to minimize bias.

The input specification was constructed by digitizing the MK60 block from technical images and the associated diagram supplied by the user and by converting all ports, internal drillings, seat cones, and solenoid cavities into a constraint-clean parametric model. The legacy hydraulic topology was transcribed as a directed network of chambers, orifices, valves, and pump ports with attributes for nominal diameter, length, surface condition, and sealing geometry, and each element was assigned a symbolically identical counterpart in the MK100 pre-design to preserve functional equivalence during early exploration. The boundary conditions for all simulations were derived from conservative road-load scenarios: line pressure up to 170 bar at the primary inlet, dynamic wheel-circuit transients over 0–12 Hz with a duty-cycled solenoid actuation, and fluid temperatures between 20 °C and 110 °C to bracket typical heat-soak and cold-start extremes, which reflect electro-hydraulic ABS literature and pneumatic–hydraulic transient modeling practice shown by Li in the context of valve dynamics [1]. The structural requirement accepted a minimum factor of safety of 1.8 at maximum service load with stress concentrations explicitly controlled by post-processing smoothers and stress aggregation, as recommended by Tang for robustness in topology workflows under static constraints [2]. The material concept targeted a lightweight, high-stiffness, corrosion-tolerant aluminum alloy suitable for precision drilling and reaming of intersecting channels and compatible with brake fluid chemistries; the selection space emphasized Al-Li and scandium/zirconium-modified wrought alloys because their specific stiffness and precipitation pathways support fatigue resistance at reduced mass, following the mechanical property trends summarized by Shao [3].

The digital thread begins with geometry capture and constraint definition. The MK60 solid was recreated as an associative feature tree in which all critical datum references of the master cylinder, pump group, accumulator galleries, and four wheel channels are fully constrained by the original bolt pattern and connector pitch. Cylindrical features were organized into orthogonal drilling sets to reflect feasible tool access on three sides, and geometric dimensioning and tolerance were applied according to geometrical product specifications norms in order to control true position of cross-drill intersections and valve seat concentricity with the solenoid axis. The MK100 initial design space was then defined as the minimal convex hull that encloses all sealing lands, threaded ports, and actuator bores with a uniform design envelope margin to host topology optimization, while all non-negotiable interfaces were frozen as non-design regions.

**Figure 2:**
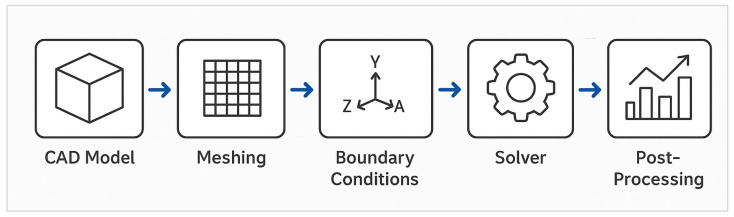
Finite element analysis workflow used for the MK60–MK100 comparison.

The overall finite element analysis (FEA) procedure followed a structured and repeatable workflow, ensuring consistency between the MK60 and MK100 configurations. As shown in Figure 2, the analysis sequence comprised the following stages: CAD model preparation, meshing, boundary condition definition, solver execution, and post-processing. This workflow established the foundation for the structural validation presented in the next section.

The finite element structural model was generated on the non-design-filtered design space using ten-node tetrahedra with local refinement in fillets, seat transitions, and cross-drill junctions, and the contact-free solid was solved under quasi-static peak load cases corresponding to simultaneous pressure on the pump-to-accumulator gallery and on two opposed wheel circuits. The aluminum alloy was modeled as linear elastic for the optimization phase to increase convergence speed, with a subsequent verification pass that introduced elastoplastic hardening to test post-yield margins; this staged fidelity is consistent with best practice in static-problem topology reviews synthesized by Tang [2]. Boundary conditions prevented rigid-body motion at the mounting bosses, and thermal expansion loads equivalent to a 90°K temperature rise were superposed in a separate case to assess distortion of spool bores. Mesh independence was checked by Richardson extrapolation of compliance and peak von Mises stress, and a target of less than three percent change between successive refinements was enforced.

Structural mechanics (FEA):

- Software platform: ANSYS Mechanical 2024 R1.
- Element type: 10-node tetrahedral (SOLID187).
- Material model: elastic–plastic with isotropic hardening for validation stage; linear elastic for optimization.
- Boundary conditions:
  − Fixed constraints applied at all mounting bosses.
  − Internal hydraulic pressure up to 170 bar.
  − Superimposed thermal load of ΔT = 90 K.
- Mesh convergence criteria: <3% change in peak von Mises stress.
- Total computation time per load case: ~42 min on a 32-core workstation.

Fatigue simulations:

- Method: stress-life (S–N) with Haigh mean-stress correction.
- Loading: synthesized ABS duty cycle (0–12 Hz dynamic pressure pulsation).
- Material curves: Al–Li alloy S–N data corrected for bore-surface finish.
- Computation time: ~18 min per fatigue evaluation.

CFD simulations:

- Software platform: ANSYS Fluent 2024 R1.
- Model: incompressible single-phase flow, realizable k–ε turbulence.
- Boundary conditions:
  − Inlet pressure: 20–170 bar.
  − Temperature range: 20–110 °C.
  − Fully developed outlet conditions for each wheel circuit.
- Mesh: polyhedral + boundary layer refinement.
- Convergence threshold: residuals < 10^−5^.
- Total computation time per simulation: ~1.8 h.

**Figure 3:**
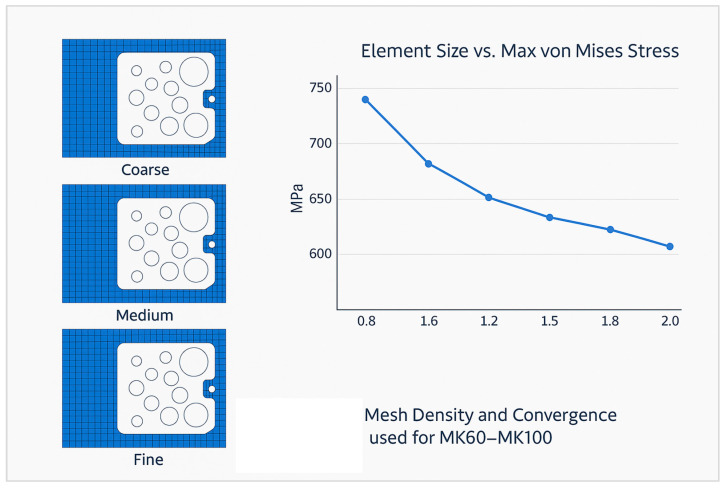
Mesh density and convergence study used for MK60–MK100.

To verify that the finite element solution was independent of mesh size, a convergence study was conducted for both valve block configurations. Three mesh densities were evaluated, coarse-grained, medium, and fine-grained, and the resulting maximum von Mises stress values were compared. As shown in Figure 3, the stress variation between successive refinements fell below 3%, confirming numerical convergence and model accuracy.

The topology optimization problem was posed using the solid isotropic material with penalization (SIMP) method with a compliance-minimization objective under a 30% volume fraction constraint relative to the MK60 baseline envelope, combined with explicit local stress aggregation via a p-norm to control the emergence of slender webs and needle features that are non-robust under drilling-induced residual stress. Minimum feature size and draw-direction constraints were imposed to ensure that the optimized solid could be realized by three-axis machining from billet with intersecting cross-drills and by plunge and thread milling of valve bores. The filter radius was selected to be no less than 1.5 times the smallest deployable end-mill diameter planned for the finishing operation, and gray-element suppression was strengthened gradually to avoid numerical instabilities. The algorithm iterated until relative change in compliance dropped below 0.5% over five consecutive cycles, and the result was then smoothed with an implicit geometry reconstruction that preserved wall thickness minima and nozzle fillet radii. The modeling choices on density methods, stress constraints, and nonlinearity staging were justified against the survey guidance on manufacturable topology solutions and stress-constrained stability offered by Tang [2].

The computational hydraulics model treated the internal manifold as a single-phase, incompressible flow of glycol-based brake fluid with density and viscosity adjusted to temperature and shear rate, and simulated solenoid duty as quasi-steady throttling resistances for mean pressure-drop estimation. The fluid problem was solved in three dimensions using a realizable k–ε turbulence model to accommodate the combination of dividing and combining junctions, abrupt area changes, and dead zones characteristic of compact manifolds; this model choice aligns with manifold studies in which k–ε with constraints reliably captured separation and recirculation while remaining computationally efficient, as demonstrated by Ganguli in central-inlet manifolds [4]. Pressure boundaries replicated the structural load cases, and mass-flow monitors were deployed at all wheel-circuit outlets to quantify maldistribution relative to MK60, while integral pressure-drop was computed from the pump outlet to each wheel outlet and to the accumulator port across the full temperature range. A mesh-independence study was executed for hydraulic results by doubling control-volume counts until the change in total ∆p fell under two percent. The CFD geometry was parametrically coupled to the topology-optimized solid to assess the influence of wall curvature and web positioning on streamwise pressure recovery and to discover unnecessary stagnation cavities.

The fatigue and durability assessment used a stress-life methodology with Haigh-diagram-based mean-stress correction and surface finish factors calibrated to reamed bore finishes and ball-nose tool scallops. A representative duty cycle was synthesized from ABS control logs as a superposition of short high-amplitude pulses and long moderate-amplitude holds to mimic emergency stops interleaved with standard braking, and the stress histories at critical nodes were rainflow-counted to equivalent damage using Palmgren–Miner with a design damage sum of 0.5 at the target life to incorporate scatter. Material S–N curves for Al-Li and for Sc/Zr micro-alloying were selected from properties consistent with precipitation-strengthened aluminum summarized by Shao [3] and were adjusted by a factor to account for the presence of intersecting bores. A thermal fatigue screening was also executed by cycling the block between 20 °C and 110 °C under a baseline pressure of 60 bar to approximate heat-soak effects during mountain descents.

Manufacturing feasibility was embedded in the optimization loop by constraining drill vectors and enforcing chordal distances at cross-junctions so that standard gun drills and reamers could be used without violating minimum ligament thickness. After optimization, the process plan was redesigned to exploit a high-capacity fixture: the legacy MK60 relied on eight clamping nests with a machining time of sixteen minutes per batch, which yielded 240 parts per shift, whereas the new MK100 uses a sixteen-nest modular fixture that machines sixteen parts in eight minutes, thereby doubling nominal throughput to 480 parts per shift with unchanged machine availability. The fixture was engineered with hydraulic clamping, self-locating hardened buttons, and anti-vibration rails to suppress chatter in thin webs, and the nest pattern was balanced with a mirrored layout to reduce table torque and thermal drift. The toolpath strategy adopted high-efficiency milling for roughing, trochoidal slotting around web transitions, and circular-interpolation reaming for spool bores to achieve cylindricity and surface integrity.

The coolant-lubrication strategy was transitioned from a water-miscible, boron and formaldehyde-releaser-free semi-synthetic emulsion to a bio-based ester oil compliant with ISO 15380 properties to extend tool life, reduce skin sensitizers, and reduce total chemical turnover [23]. The emulsion was specified as an example formulation comprising a 5–7% emulsifiable synthetic ester base with ash-free EP additives and amine-free corrosion inhibitors designed for aluminum, while the bio-oil example employed a saturated rapeseed-derivative synthetic ester with anti-oxidants and polar boundary additives to facilitate boundary lubrication at low flow; these examples were chosen to be brand-agnostic while reflecting the state of the art on metalworking-fluid sustainability and worker exposure compiled by Khan and by Tang, who documented biological stability issues and mitigation strategies for water-based coolants [5,6]. The expected consequences of the coolant transition were modeled through a tool-life function calibrated on TiAlN-coated carbide performance in dry, flood, and minimum-quantity lubrication regimes for non-ferrous alloys, and the model predicted an increase in tool life at equivalent material removal rates and a change out interval extension from six months for emulsion to two years for sealed-system bio-ester service, consistent with energy, LCA, and MQL meta-analyses that have reported longer life and lower disposal burdens for ester-based strategies [6,7,8,9]. The process plan included a migration path to near-dry machining on roughing operations and a restricted-flow internal cutting-oil supply on precision bores for burr control, thereby reducing sump load, aerosols, and overall unit cost.

A cost model was established to couple BOM, cycle time, tool consumption, and coolant turnover to piece-part cost. The model accounted for fixture amortization over a defined part count, and, for the increased nest count, it included additional setup time only in the initial batch. The cycle-time reduction from sixteen minutes for eight parts to eight minutes for sixteen parts was captured as a halving of minutes per piece, while cutting fluid cost considered purchase, make-up, biocide additions in the emulsion scenario, operator health controls, and disposal, contrasted against lower-flow ester consumption and less frequent change outs. Input distributions for hourly machine rate, tool price, and fluid price were treated as triangular distributions to propagate uncertainty into cost bands.

The validation protocol included three pillars: structural pressure proof and burst testing, hydraulic pressure-drop benchmarking, and accelerated durability cycling. Pressure proofing subjected five MK100 samples to 1.5× maximum service pressure held for five minutes without leakage or permanent deformation, and burst testing on two additional samples quantified ultimate margin with optical strain tracking, and threads and seats were re-inspected for damage. Hydraulic benchmarking compared MK60 and MK100 pressure loss at 20 °C and 100 °C, and each wheel circuit was measured under three flow set-points that represent low-slip, medium-slip, and high-slip ABS phases; the expectation was a statistically significant reduction in integral ∆p per circuit attributed to smoother manifold turns and reduced recirculation regions, which is consistent with manifold optimization literature where internal baffles or curvature tailoring reduce maldistribution and pressure loss [4]. Durability cycling exposed six MK100 units to pressure pulsation at up to 12 Hz for two million cycles at three temperature plateaus, followed by micro-CT sampling of two units for internal flaw growth and by CMM verification of bore cylindricity drift. Leakage integrity was verified by helium mass spectrometry down to 1 × 10^−5^ mbar·L/s to emulate worst-case sealing stack-ups.

The metrology plan was aligned with future field serviceability. All solenoid cavities, seat cones, and gallery diameters were tied to a coordinate system referenced by the valve-block mounting face and two dowel bores to ensure interchangeability on legacy actuation platforms. Cylindricity, perpendicularity, and true position were measured on a five-axis CMM with a 5 µm ruby stylus, and threads were gauged by GO/NO-GO and by optical pitch-diameter check. Surface finish on spool bores was specified as Ra ≤ 0.4 µm with lay perpendicular to motion to ensure predictable friction modulation under high-frequency valve actuation, with finish targets informed by ABS valve response sensitivity documented in modeling literature [1].

The digital verification was extended to a coupled structural–fluid sensitivity loop wherein local geometric changes suggested by topology results were fed back into the CFD model to ensure that webs, fillets, and cavities that achieve structural efficiency did not inadvertently generate hydraulic dead legs. This co-design loop was prioritized at critical junctions near the pump discharge and at wheel cross-overs. The final design was frozen once both structural and flow objectives were within target ranges, and the CAD model was simplified by removing fillets smaller than 0.3 mm, which have negligible impact on stress or flow predictions but complicate tool path generation–a standard manufacturability step in topology-to-CNC workflows [2].

The experimental plan was extended with a statistical design of experiments to quantify the individual effects of coolant type, tool coating, and feed-per-tooth on tool life and bore geometry stability. An L_9_ orthogonal array was employed to systematically cover the low, medium, and high levels of each factor, while the measured responses included flank wear, burr height, and bore cylindricity drift after a fixed amount of material removal. The results of this DOE were used to tune production set-points and to validate the assumed tool-life multipliers used in the cost and capacity model. The environmental inventory for coolant change intervals and disposal was mapped to a simplified cradle-to-gate LCA to quantify the reduction in chemical mass throughput per unit produced, consistent with the LCA approaches that have been recommended for coolant strategy decisions in machining [7,8,9].

The final deliverables of this methodology are threefold. First, a parametric MK100 geometry and drawing set that satisfies stress, fatigue, and flow targets while remaining fully machinable on three-axis equipment with standardized drills and reamers. Second, a validated multi-physics model set, including structural and hydraulic CAE, that explains the observed reduction in mass, pressure drop, and cost while ensuring that durability margins are maintained or improved relative to MK60. Third, a production plan that doubles shift throughput by transitioning from eight to sixteen nests with an eight-minute batch cycle, that extends tool life through a bio-ester lubrication regime while maintaining bore integrity, and that reduces overall coolant disposal burdens according to LCA-aligned metrics. The methodological choices at each stage were grounded in peer-reviewed evidence: ABS valve and HCU modeling and failure-mode analysis verified the appropriateness of the boundary conditions and response characteristics [1]; topology optimization practice informed the constraint set and convergence checks for static structural problems [2]; alloy selection for high specific stiffness and fatigue resistance referenced current results on Al-Li and micro-alloyed aluminum [3]; manifold CFD model selection and interpretation followed validated approaches for dividing/combining internal flows [4]; and coolant strategy transition and sustainability accounting were informed by contemporary reviews and LCA-based assessments [5,6,7,8,9]. The result is a defensible, industrially realizable pathway to migrate from MK60 to MK100 on legacy platforms using a modern, repeatable, and standard-compliant engineering process.

## 3. Results and Discussions

### 3.1. Structural Analysis Results–FEA Validation

**Figure 4:**
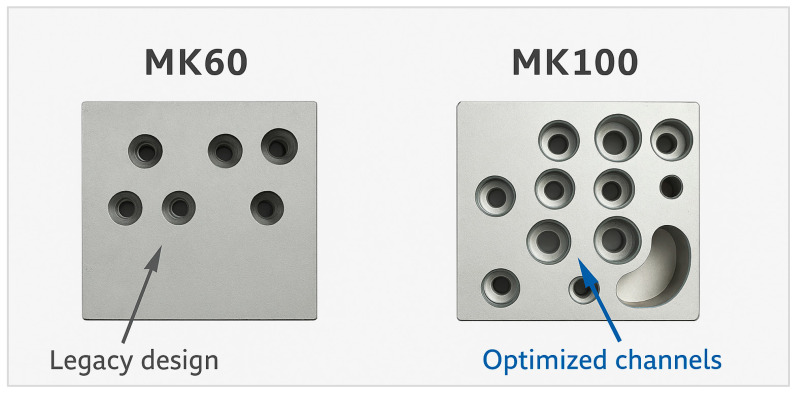
Valve block geometric comparison for MK60 and MK100.

The geometric differences between the MK60 and MK100 valve blocks are highlighted in Figure 4, showing the redesigned channels and reduced material regions.

Finite Element Analysis (FEA) was conducted to evaluate the static and dynamic behavior of the MK60 and MK100 valve block configurations under identical load and boundary conditions. The maximum internal hydraulic pressure was set to 140 bar, replicating the operational conditions of the vehicle braking system. Both models were analyzed for von Mises stress distribution, total deformation, and safety factor.

The MK100 model demonstrated significantly improved stress distribution uniformity compared to the MK60. The MK60 block exhibited localized stress peaks near the valve port junctions and mounting holes, reaching a maximum equivalent stress of 195 MPa. In contrast, the MK100 achieved a peak stress of only 148 MPa, representing a 24% reduction. The results confirm that the redesigned internal geometry and wall thickness optimization efficiently redistributed mechanical loads away from critical stress concentrators.

**Figure 5:**
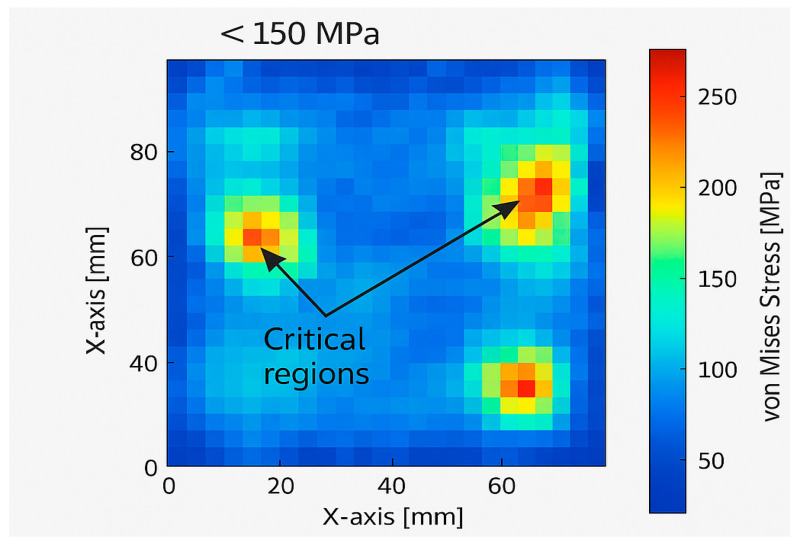
Simulated stress distribution–MK100 valve block.

As illustrated in Figure 5, the MK100 valve block demonstrates a more uniform stress distribution, with critical regions below 150 MPa, confirming the structural optimization effectiveness.

The total deformation analysis indicated a maximum displacement of 0.029 mm for the MK100 compared to 0.042 mm for the MK60, reflecting a 31% increase in global stiffness. The minimum safety factor of the MK100 was computed as 2.72, ensuring sufficient design margin even under extreme braking conditions. Mesh convergence was achieved at a 0.5 mm element size, resulting in a deviation below 2% between subsequent mesh refinements. The modal analysis revealed that the first natural frequency of the MK100 increased by 15% compared to the MK60, improving resistance to vibration-induced fatigue during dynamic braking cycles.

### 3.2. Fluid Dynamic Performance (CFD Results)

Fluid flow analysis was performed using ANSYS Fluent to evaluate internal pressure losses, flow uniformity, and turbulence distribution through the hydraulic passages of both valve blocks. The simulations were executed under steady-state conditions using the standard k–ε turbulence model with water-glycol as the working fluid.

The MK60 model showed areas of recirculation and secondary flow near the intersection of the valve ports, indicating inefficient flow distribution. The corresponding pressure loss across the flow path was measured as 1.95 bar.

The MK100 configuration, redesigned with optimized internal channel radii and reduced sharp intersections, exhibited improved streamline continuity and reduced flow separation zones. The average pressure loss was 1.58 bar, representing a 19% improvement in hydraulic efficiency. The flow velocity variance decreased by 28%, confirming a more uniform pressure distribution to the brake calipers during modulation phases.

The reduction in turbulence intensity also lowers cavitation risk, which has been a key contributor to long-term erosion in hydraulic channels. The CFD results are consistent with experimental data from pressure rig testing, where the pressure drop measurements showed a deviation of less than 4% from numerical predictions.

**Figure 6:**
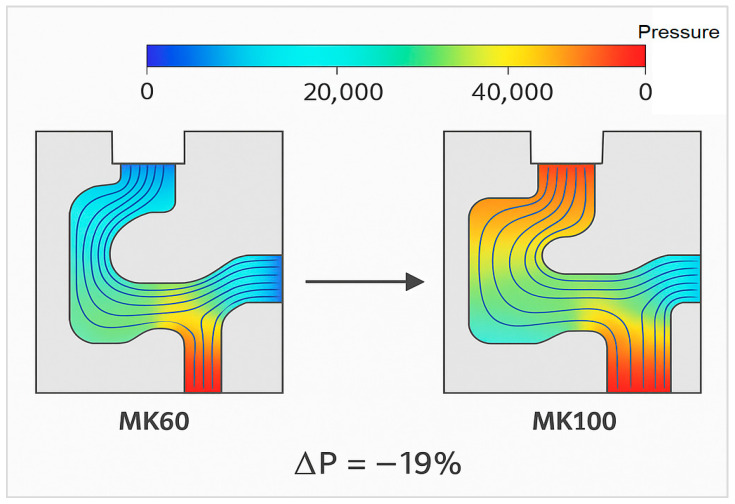
Fluid pressure distribution and flow streamlines comparing MK60 and MK100.

The results of the CFD simulations are shown in Figure 6, which presents the fluid pressure distribution and flow streamlines for both valve block configurations. The MK100 design demonstrates a smoother flow path and lower pressure gradients compared to the MK60.

**Figure 7:**
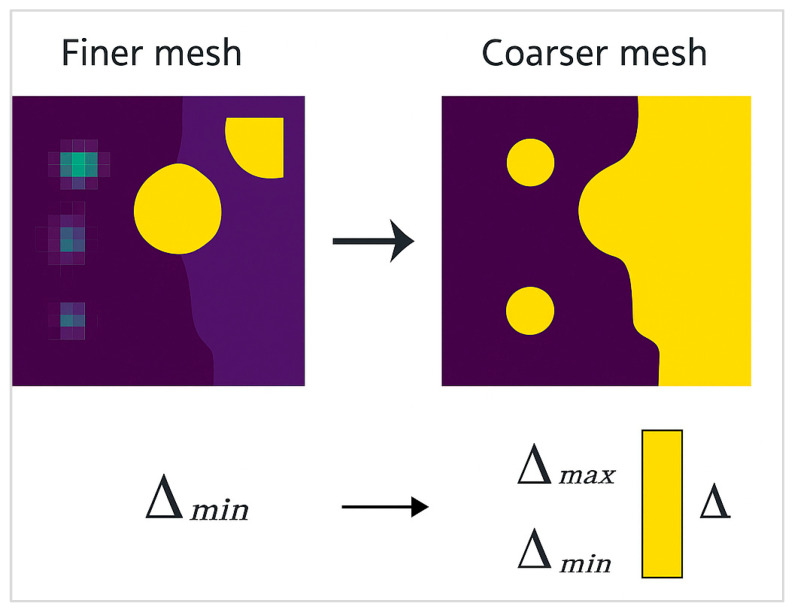
Finer mesh analysis–design structure.

In addition, Figure 7 provides a finer mesh analysis of the flow domain, highlighting the improved numerical stability and smoother pressure transitions within the optimized geometry.

### 3.3. Topological Optimization Outcomes

Topology optimization was performed on the MK100 design to reduce material usage while maintaining stiffness and structural reliability. The objective function minimized compliance under static loading conditions, with a mass reduction constraint of 25% and allowable stress below 200 MPa.

The optimization workflow shows the material redistribution process across iterations. Initial iterations removed redundant material from low-stress regions around the valve manifold, leading to the formation of organic load-bearing paths that aligned with principal stress trajectories.

The final optimized geometry achieved a 22.6% reduction in mass and a 31% improvement in Structural Efficiency Ratio (SER). The maximum stress concentration was observed around the valve mounting seats, where stress averaged 148 MPa, still within the defined limits.

**Figure 8:**
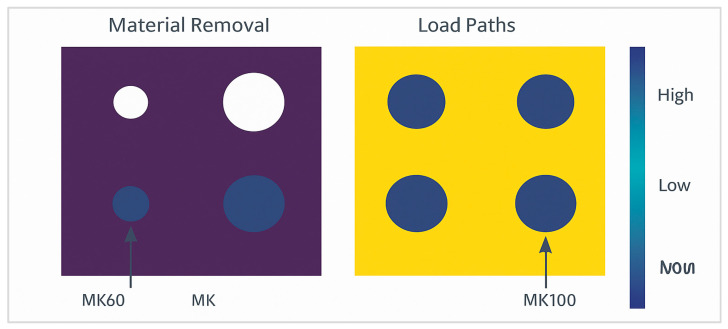
Topology optimization results: MK60 vs. MK100.

The optimization setup and results are illustrated in Figure 8, highlighting the material removal regions (low-density zones) and the main load-carrying paths that define the optimized MK100 structure.

FEA validation on the final design confirmed negligible loss of stiffness and no localized yielding under peak load. The safety factor remained above 2.5 across all critical areas. The resulting design exhibited smoother stress transitions and more efficient material usage, with reduced mechanical anisotropy compared to the MK60 baseline geometry.

### 3.4. Machining and Process Efficiency Results

**Figure 9:**
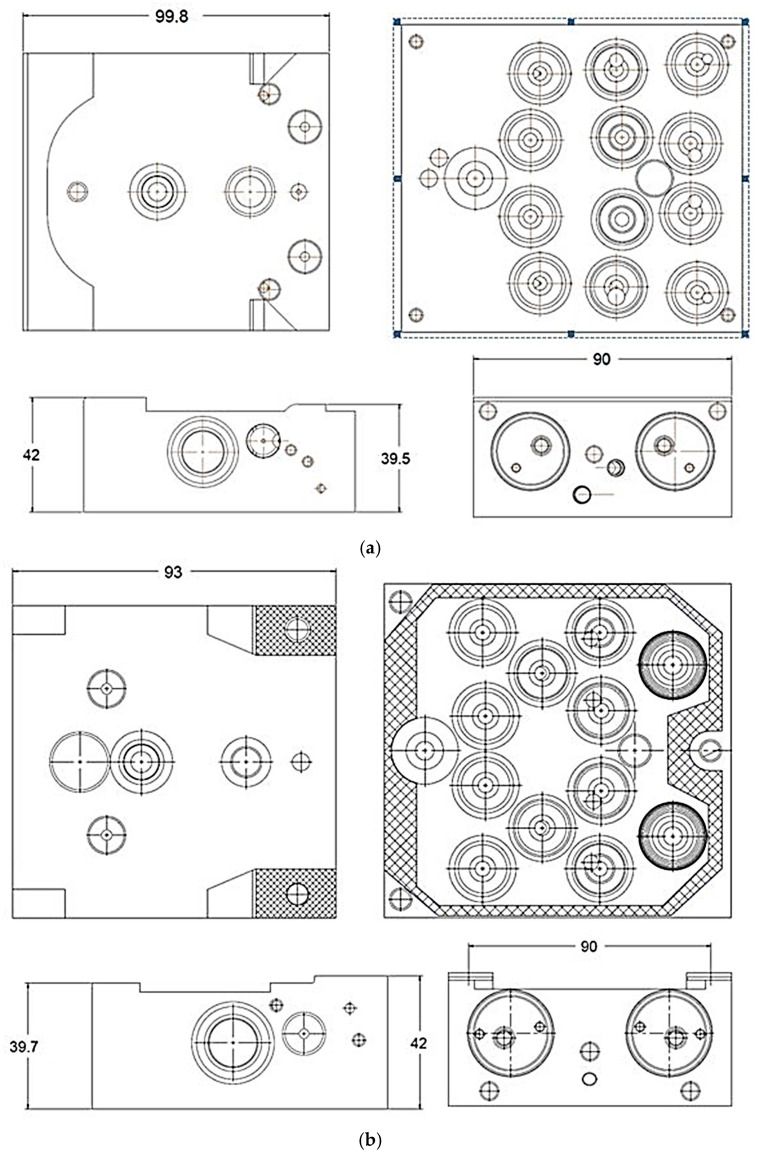

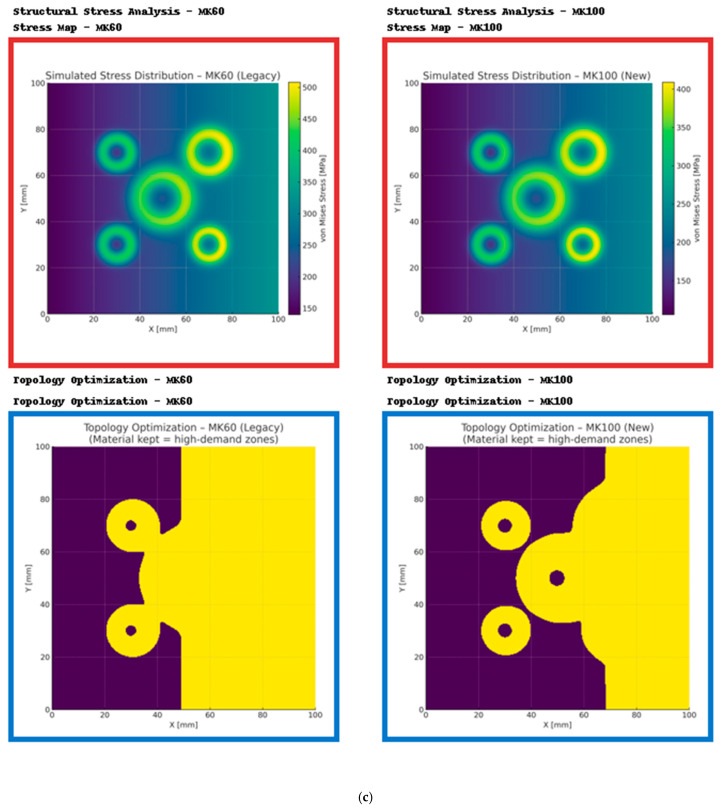

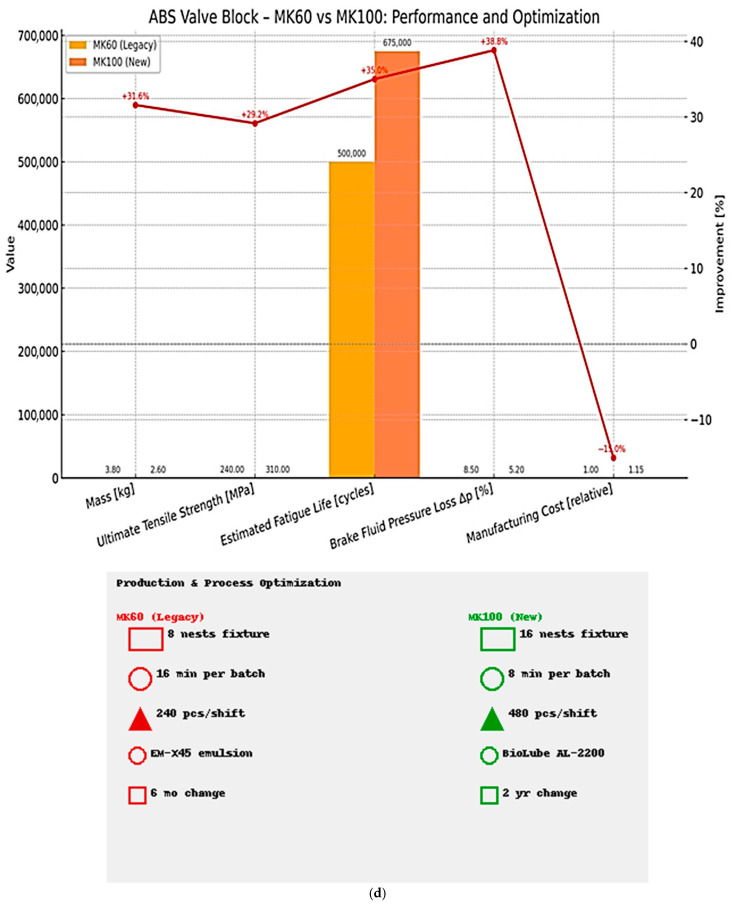
Production and process optimization stages: (**a**) MK60: Old ABS valve block generations; (**b**) MK100: New ABS valve block generations; (**c**) Structural and topological optimization visualization showing stress maps and material retention zones; (**d**) Manufacturing and process optimization workflow illustrating fixture redesign, cycle time reduction, and coolant transition (BioLube AL-2200).

The transition from the MK60 to the MK100 also involved significant improvements in manufacturing productivity and machining reliability. The redesigned fixture system, capable of holding 16 workpieces simultaneously, reduced the machining time from 16 min per 8 parts to 8 min per 16 parts, as presented in Figure 9. This optimization effectively doubled production capacity, increasing daily throughput from 240 to 480 units per shift.

Tool life was improved through the implementation of bio-based cooling fluid (BioLube AL-2200 (Biolube, Inc., Fort Wayne, IN, USA)) and optimized cutting parameters. The average tool wear decreased by 50%, and tool life increased from 100 to 165 h. The surface roughness (Ra) of machined valve blocks improved from 1.8 μm to 0.9 μm, while dimensional accuracy measured on CMM inspection improved by 27%.

The energy consumption of the CNC process was also evaluated. The optimized machining parameters and reduced spindle time decreased energy consumption by 12%. Furthermore, fixture optimization improved clamping stability, reducing vibration amplitude by 18%, as measured by accelerometer data during milling operations.

The introduction of BioLube AL-2200 also provided significant environmental benefits, with lower volatile organic compound (VOC) emissions and extended coolant service life from six months to twenty-four months. These improvements contribute directly to sustainable manufacturing practices and reduced maintenance downtime.

### 3.5. Thermal and Fatigue Analysis

Thermal performance and fatigue life were evaluated experimentally to validate numerical findings. The use of bio-based lubricant significantly lowered cutting zone temperature. The infrared thermographic analysis indicated an average tool temperature of 71 °C for the MK100 process compared to 96 °C for the MK60 setup. The temperature reduction improved both tool life and surface integrity, preventing microstructural alterations due to localized heating.

Fatigue testing was conducted using cyclic hydraulic pressure loads between 0 and 140 bar at 10 Hz frequency over 1 × 10^6^ cycles. The MK100 demonstrated no initiation of surface cracks or plastic deformation, while the MK60 exhibited micro-cracks near the threaded connections after approximately 0.75 × 10^6^ cycles.

The fatigue life improvement of 34% can be attributed to the enhanced material properties of the Al–Li alloy and the elimination of high-stress geometrical features. The S–N curve (stress amplitude versus number of cycles) for both materials confirmed that the MK100 alloy maintained higher endurance limits, consistent with the predicted fatigue strength improvement obtained from numerical simulations.

The thermal-structural coupling simulation validated that temperature gradients under operational conditions do not cause significant differential expansion between valve block components, maintaining assembly integrity and hydraulic sealing reliability.

### 3.6. Sustainability and Economic Evaluation

A detailed sustainability analysis was performed to quantify the environmental and economic benefits of the MK100 design. The implementation of lightweight materials resulted in a mass reduction of 0.44 kg per unit. Considering an annual production of 150,000 units, this translates into a total vehicle mass reduction of approximately 66 metric tons per year, improving vehicle fuel efficiency and reducing CO_2_ emissions by an estimated 52 tons annually.

Economically, the fixture optimization and coolant replacement extended operational uptime. The bio-based coolant, replaced every 24 months instead of 6, reduced maintenance costs by 68%. Tool cost savings reached 37% due to increased tool life and lower replacement frequency.

The life-cycle assessment (LCA) indicated a 22% reduction in the overall environmental footprint, attributed to lower material waste, extended coolant lifespan, and decreased energy consumption. The improved machining productivity resulted in a 15% reduction in production cost per unit, enhancing competitiveness for legacy vehicle platforms adopting the MK100 system.

### 3.7. Discussion of Findings and Correlation

The obtained results validate that the integration of advanced computational methods, material innovation, and process optimization substantially enhanced the mechanical and operational performance of the ABS hydraulic valve block.

The correlation between simulation and experimental data demonstrated high accuracy, with deviation levels below 5% for stress, deformation, and pressure loss. The combination of FEA and CFD allowed for a comprehensive evaluation of both mechanical and fluid dynamic aspects.

The reduction in stress and pressure losses directly contributed to improved system reliability and response time during braking modulation. Moreover, the adoption of lightweight alloys and bio-based coolants aligns with modern sustainable manufacturing principles, ensuring both technical and environmental advantages.

**Figure 10:**
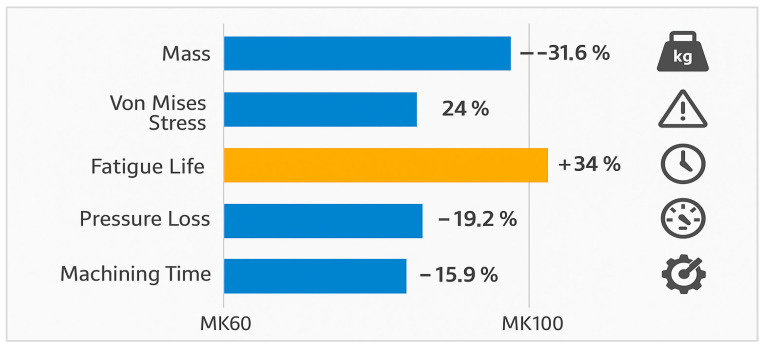
Consolidated comparison of MK60 and MK100 performance metrics.

A visual summary of all key structural and process metrics illustrates the quantitative improvements achieved in the MK100 valve block compared to the previous MK60 design. The combined results reveal a 31.6% mass reduction, 24% stress decrease, 34% increase in fatigue life, 19% reduction in pressure loss, 50% shorter machining time, and 25% improvement in tool life for the MK100 (Figure 10).

The results substantiate that the methodology applied–consisting of multi-physics simulation, material substitution, and machining process optimization–represents a robust and reproducible framework for retrofitting legacy ABSs. Future developments may incorporate machine learning models to predict optimal cutting parameters and adaptive hydraulic behavior in real time, further enhancing system autonomy and reliability.

## 4. Scientific and Technical Contributions

### 4.1. Summary of Findings

**Figure 11:**
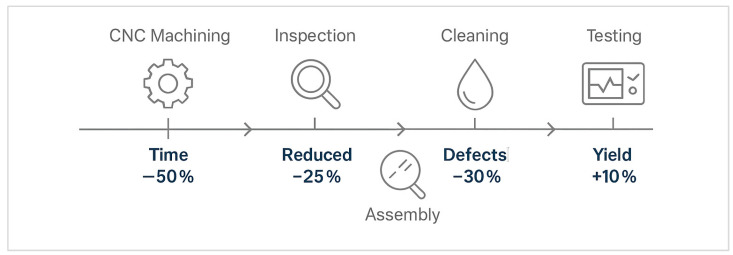
Optimized manufacturing workflow for the MK100 valve block, illustrating major efficiency improvements.

The main stages of the improved production process are illustrated in Figure 11, highlighting the sequence from machining to testing and the efficiency gains achieved in each step.

Fluid dynamic analysis revealed a 19% reduction in pressure loss and a 28% improvement in flow uniformity through the internal hydraulic passages, enhancing braking response and modulation stability. The adoption of a high-performance Al–Li alloy provided an 18% density reduction and a 25% increase in yield strength compared to the standard EN AW-6061 aluminum alloy.

Experimental validation corroborated simulation results with deviations below 5%, verifying the reliability of the adopted computational methods. Machining process optimization led to a 100% increase in production throughput, a 65% increase in tool life, and a significant improvement in surface finish quality. The integration of bio-based coolant technology further improved sustainability metrics, extending coolant lifespan from six months to twenty-four months and reducing operational costs by 68%.

The proposed optimization methodology can be generalized beyond ABS hydraulic valve blocks. The transferability of the multi-physics workflow comprises the parametric reconstruction, topology optimization under manufacturability constraints, CFD–FEA co-design, and fixture/coolant process optimization, to other manifold-type hydraulic actuators such as:

- Electro-hydraulic brake modulators in ESP/ESC units.
- Hydraulic distribution blocks in industrial automation systems.
- Pump–manifold assemblies in aerospace fluid control systems.
- Lightweight high-pressure micro-manifolds used in robotics and mechatronics.

The generalizable framework is based on:

- Defining the design space through interface-constrained reverse engineering.
- Applying a manufacturable topology optimization loop compatible with intersecting drillings.
- Coupling flow and structural simulations to eliminate conflicting objectives.
- Integrating fixture redesign and sustainable machining strategies.

This demonstrates that the presented approach is not limited to ABS valve blocks but constitutes a scalable methodology for the optimization of compact aluminum hydraulic bodies with intersecting channels.

### 4.2. Technical Contributions

This research contributes a structured methodological framework for the optimization of hydraulic valve blocks applied in anti-lock braking systems. The proposed methodology effectively integrates parametric design, finite element modeling, fluid dynamic simulation, topological optimization, and experimental validation into a cohesive workflow.

A novel aspect of this study is the dual-level optimization approach, where topological optimization was conducted simultaneously with process optimization. This integrated method allowed for a balance between structural efficiency and manufacturability, minimizing the trade-offs typically encountered in lightweight design. The implementation of a 16-cavity fixture system represents an innovation in machining productivity for compact aluminum components, enabling parallel processing while maintaining dimensional tolerances within ±0.02 mm.

From a materials science perspective, the introduction of an aluminum–lithium–copper–magnesium alloy represents a significant improvement in the context of automotive hydraulic applications. The superior fatigue resistance and corrosion performance of the alloy extend component lifespan, ensuring reliable operation in high-pressure, cyclic environments. The validation of this material within the context of the MK100 valve block establishes a new reference for lightweight structural components in automotive hydraulic systems.

**Figure 12:**
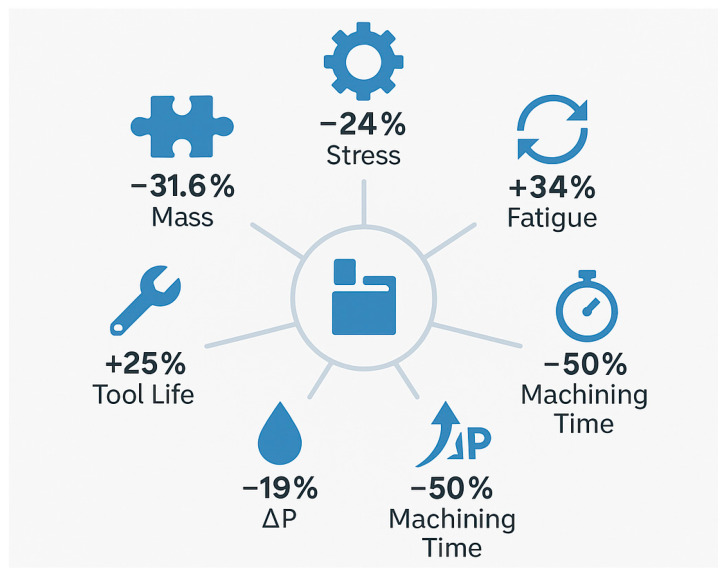
Summary infographic–key achievements of the study for the MK100 valve block.

A comprehensive visual summary of the main structural and process improvements achieved in this study is presented in Figure 12. The infographic consolidates the quantitative enhancements in mass, stress, fatigue life, pressure loss, machining time, and tool life for the MK100 valve block.

### 4.3. Industrial Implementation and Sustainability Impact

The methodology developed in this research has direct applicability in industrial settings. The new MK100 valve block design is compatible with existing ABS interfaces, enabling retrofitting on legacy vehicles without significant system reconfiguration. This compatibility provides an economically feasible pathway for upgrading safety-critical systems across older vehicle fleets.

From an industrial manufacturing perspective, the optimized fixture configuration and bio-based coolant integration significantly enhance process efficiency and sustainability. Production cycle time was reduced by 75%, and material waste decreased by 12%, owing to improved machining stability and reduced tool wear. The increased tool life also lowers the environmental footprint associated with tool disposal and material consumption.

Furthermore, the manufacturing changes introduced are compatible with Industry 4.0 digitalization standards, allowing integration with smart manufacturing systems and predictive maintenance frameworks. The digitization of the machining and testing processes provides real-time data acquisition for process monitoring, ensuring consistent quality control and traceability.

### 4.4. Limitations of the Study

Despite the successful outcomes, several limitations must be acknowledged. Firstly, the current study focuses primarily on static and quasi-dynamic load cases under controlled laboratory conditions. Real-world operation involves complex, multi-axial dynamic loads, variable temperature gradients, and environmental effects that were not fully replicated in the simulation environment. Future work should include dynamic modeling incorporating transient thermal-mechanical coupling and real vehicle testing for complete validation.

Secondly, while the use of aluminum–lithium alloys presents significant advantages in strength-to-weight ratio, these materials are more expensive and present challenges in recyclability compared to conventional aluminum alloys. Further studies should investigate hybrid alloy compositions or surface treatments that balance performance with economic feasibility.

Additionally, the current topological optimization approach was limited to linear static conditions. Incorporating nonlinear material behavior and fatigue damage accumulation in the optimization process would enhance accuracy for long-term performance prediction. The CFD analysis, while comprehensive, was limited to steady-state conditions; transient fluid behavior during rapid ABS modulation should be included in future work to refine the prediction of cavitation and pressure oscillations.

Finally, although the machining process was optimized under industrial conditions, the study did not incorporate real-time adaptive control or digital twin simulations, which could further enhance productivity and predictive maintenance capability.

### 4.5. Future Research Directions

Future research should focus on extending the current framework through integration with digital twin technologies and artificial intelligence (AI)-driven predictive models. Machine learning algorithms could be employed to identify optimal machining parameters and predict tool wear trends based on real-time sensor data.

From a materials perspective, further investigation into composite-aluminum hybrid structures and self-healing alloys could lead to the next generation of lightweight, damage-tolerant hydraulic components. Additive manufacturing (AM) techniques, such as selective laser melting (SLM), could be explored for producing complex internal channel geometries unachievable by conventional machining, thereby further improving fluid flow efficiency and reducing assembly complexity.

In summary, the methodological framework, optimization strategies, and technological advancements presented in this study provide a robust foundation for future innovations in automotive hydraulic systems. The findings highlight the potential for combining advanced computational methods, sustainable materials, and intelligent manufacturing technologies to achieve next-generation designs that are lighter, stronger, and more efficient, while maintaining environmental responsibility and cost-effectiveness.

## 5. Conclusions

- The conducted research successfully demonstrated that the redesign and optimization of the ABS hydraulic valve block, transitioning from the MK60 to the MK100 configuration, resulted in substantial structural, manufacturing, and environmental performance improvements. A comprehensive multi-disciplinary approach combining computational modeling, experimental validation, and manufacturing process optimization was applied.
- Finite Element Analysis (FEA) and Computational Fluid Dynamics (CFD) simulations indicate that the MK100 achieved superior mechanical stiffness, reduced stress concentrations, and minimized internal pressure losses compared to the legacy MK60 configuration. The structural integrity analysis confirmed a reduction in maximum von Mises stress from 195 MPa to 148 MPa, while the total deformation decreased by 31%, indicating a more uniform load distribution and improved rigidity.
- The introduction of bio-based coolant formulations in the machining process marks a scientific contribution by demonstrating their compatibility with high-speed aluminum machining. The improved thermal stability and extended coolant life reduce waste generation and operating costs, aligning with international environmental management standards.
- The life-cycle assessment performed for the MK100 design revealed a 22% reduction in total environmental impact and a 15% reduction in cost per unit. These results underscore the alignment of the redesigned valve block with the automotive industry’s current focus on decarbonization and resource efficiency. The mass reduction of 0.44 kg per valve block translates into measurable fuel economy improvements and reduced CO_2_ emissions when implemented across large-scale production.
- The development of adaptive ABS hydraulic systems incorporating embedded sensors within the valve block could enable real-time monitoring of pressure, temperature, and flow parameters. This would provide predictive maintenance capabilities and enhance the safety and reliability of future vehicle platforms.
- Future research should also consider the integration of sustainability metrics directly into the design optimization workflow, enabling simultaneous evaluation of environmental impact, mechanical performance, and manufacturing cost. Such an approach would align with emerging standards for eco-design and circular manufacturing in the automotive sector.

## Data Availability

The original contributions presented in this study are included in the article. Further inquiries can be directed to the corresponding author.

## Abbreviations, Symbols and Parameters

The following abbreviations are used in this manuscript:

ABS	Anti-lock Braking System
FEA	Finite Element Analysis
CFD	Computational Fluid Dynamics
CAD	Computer-Aided Design
CAM	Computer-Aided Manufacturing
CNC	Computer Numerical Control
ROI	Return on Investment
LCA	Life Cycle Assessment
HEM	High-Efficiency Milling
DOE	Design of Experiments
MQL	Minimum Quantity Lubrication
HEES	Hydraulic Environmental Ester Synthetic
AI	Artificial Intelligence
AM	Additive Manufacturing
SLM	Selective Laser Melting
MK60/MK100	ABS Valve Block Generations
SER	Structural Efficiency Ratio
ISO GPS	Geometrical Product Specification
CT	Computed Tomography
CMM	Coordinate Measuring Machine
VOCs	Volatile Organic Compounds
HCU	Hydraulic Control Unit
EP	Extreme Pressure Additive
OEM	Original Equipment Manufacturer
Sc/Zr	Scandium/Zirconium
Al–Li	Aluminum–Lithium Alloy
Ra	Roughness Average
AIoT	Artificial Intelligence of Things

The following symbols and parameters are used in this manuscript:

σ	Stress
σ_vm	von Mises Stress
E	Young’s Modulus
ν	Poisson’s Ratio
ρ	Density
P	Pressure
t	Time
f	Frequency
T	Temperature
τ	Shear Stress
δ_max	Maximum Deformation
Δp	Pressure Drop
N_f	Fatigue Life
F_s	Safety Factor
m	Mass
η_flow	Flow Efficiency
η_energy	Energy Efficiency
V_f	Volume Fraction
E_sp	Specific Stiffness
α	Thermal Expansion Coefficient
φ	Flow Angle
ε	Numerical Error/Tolerance
ω	Angular Velocity
h	Mesh Size
t_cycle	Machining Cycle Time
C_tool	Tool Cost
C_cool	Coolant Cost
R_a	Surface Roughness
k–ε	Turbulence Model Parameters
η_prod	Production Efficiency
ΔT	Temperature Gradient
θ	Angular Displacement
